# Active ageing policy in challenging production environments: a case study involving social partners in Spain

**DOI:** 10.1007/s10433-021-00650-6

**Published:** 2021-09-24

**Authors:** Mariano Sánchez, Pilar Díaz

**Affiliations:** 1 Facultad de Ciencias Políticas y Sociología, Universidad de Granada, 18071 Granada, Spain; 2 Facultad de Relaciones Laborales y Recursos Humanos, Universidad de Granada, 18071 Granada, Spain

**Keywords:** Active ageing policy, Older workers, Social partners, Spain

## Abstract

In recent years, due to population ageing, the European Union has promoted active ageing policies as a means to encourage workers to delay labour market exit. Our paper explores what active ageing means to social partners (employers, managers and workers) in Spain and also the viability of implementing an active ageing culture in organizations. We used a qualitative case study methodology to identify perceptions regarding obstacles and facilitators in the adoption of an active ageing culture in different production sectors, as well as responses to active ageing policies. Data-generating workshops were held with employees and managers of four types of companies located in the south of the country. Perceived obstacles to active ageing were more organizational than individual in nature. Some of these obstacles gave rise to a kind of forced active ageing in a context in which the previous predominant message had been to retire as early as possible. Job adaptation was perceived as both an obstacle and a facilitator. Increasing recognition of older workers and raising their motivation to extend their working lives were found to be important pending tasks. In contexts of high unemployment, active ageing often remains largely unknown within productive organizations, and the idea of retiring as soon as possible continues to guide many workers. In these circumstances, the implementation of top-down, uniform active ageing policies has proven to be of limited efficacy. Awareness campaigns and initiatives aimed at improving older workers’ health and abilities should be launched as complementary active ageing strategies.

**Supplementary Information:**

The online version contains supplementary material available at 10.1007/s10433-021-00650-6.

## Introduction

The ageing of the population in Europe has led to a greater proportion of older people in today’s society. Spain is no exception to this trend (INE 2016), a result of the country’s increased life expectancy (INE 2018) and lower fertility (FBBVA 2019). This demographic transformation has naturally had an impact on the Spanish labour market, where the activity rate of people over 55 years old has increased by eleven points since 2010 (INE 2020). However, the fact that these workers are gradually reaching retirement age, together with the fact that young people are joining the labour market in smaller numbers and later in life, due to the lack of job opportunities (Rufino 2018; Velázquez 2015), means that there has been an overall decrease of working-age population and a relative increase of retired people (Eurostat 2018). Added to these dynamics is the effect of early exit from the labour market: a significant number of workers over 50 years of age have retired—voluntarily or involuntarily—from their work activity before legal retirement age (Alcover et al. 2014; OECD 2018). Currently, neither policies designed to discourage early retirement (Gutiérrez 2017) nor strategies put in place to support older workers (Martínez 2019) have produced satisfactory results; quite the opposite in fact. The periods of activity in the Spanish labour market are becoming shorter, while the retirement period is expected to last longer due to the many years that Spanish workers can expect to live after they retire (Abellán et al. 2018). In such a context, it is clear that “population aging will continue to put pressure on public finances” (OECD 2018: 7) as it is expected that the Spanish dependency rate will double between 2015 and 2050, occupying the second-highest position among OECD members (OECD 2017).

The impact of these demographic changes on the structure and capacity of the labour market, along with their socio-economic consequences, has been the subject of lively debate in Spain. One issue discussed is the need to re-examine the balance between the working period and the retirement period. The debate, which generally focuses on older workers because they are the only group in the active population that will increase over coming years (Observatorio de las Ocupaciones 2019), has led to the implementation of policies aimed at prolonging the working life. Some of these policies have been proposed under the name of active ageing policies (Spain 15 March 2013).

Active ageing can be understood in a number of different ways (OECD 2000; WHO 2002). Taking into account that European Union (EU) policies have the greatest direct impact on the cases examined in this paper, it seemed appropriate to use the European Commission (2012) understanding of active ageing (hereinafter referred to as AA). According to this institution, the meaning of AA is threefold: (1) “enabling women and men to remain in employment longer—by overcoming structural barriers and offering appropriate incentives –, (2) “facilitating active citizenship”, and (3) “enabling both women and men to keep in good health and to live independently as they grow older” (European Commission 2012: 3).

This AA paradigm includes more than just issues related to economic productivity and the labour market. Yet it has been demonstrated that a clear shift is underway towards a more production-focussed notion of active ageing (van Dyk 2015) and the EU has made statements to the effect that retirement needs to be reconsidered (López-López and Sánchez 2020). In fact, the concept of active ageing has become one of the guiding principles of labour and retirement policy in the EU (Krekula and Vickerstaff 2020) and elsewhere (Pham et al. 2020), because of the influence that demographic ageing has on the labour market. The EU recently advised Spain to take action in the area of employment opportunities for older workers, so as to improve this country’s active ageing profile (Walker 2019).

The European political agenda has pressured Member States to avoid early retirement practices, to introduce mechanisms to delay labour market exit and to promote the continued presence of older workers in the labour market, often through AA policies (Walker 2016). Over time, the connection between active ageing and the prolongation of working life has not just been assumed, it has been presented as advantageous for both society and workers (European Commission 2012), despite arguments and international evidence to the contrary. According to some researchers, “continued workforce participation might not necessarily benefit older people” (Taylor 2019: 102). Similarly, gerontologists critical of this assumption argue that retirement periods should be protected against working life extension and activation (van Dyk 2014).

In contrast with the political institutions, there are few macro-narratives regarding AA (van Dyk et al. 2013) from European social partners—employers, workers and their representatives. However, the signing in 2017 of a framework agreement by these partners (CES et al. 2017) to effectively promote active ageing has changed the scenario. This agreement understands active ageing to be about optimizing opportunities for workers of all ages to be safe and healthy and to develop their skills and employability while working, “until legal retirement age” (p. 4), therefore discourageing early retirement, in line with the European Commission’s position. The agreement states that “[its] purpose is to foster healthy and productive working lives in a life course perspective” (p. 7). It makes no mention of extending working lives beyond retirement age.

This unprecedented framework agreement has provided an opportunity to conduct research geared at strengthening the empirical foundation of governmentality studies, moving towards a systematic reconstruction of the “dispositif of active aging” (van Dyk et al. 2013). While there is considerable information about what international institutions and governments are doing in this regard, little is known about how social partners are receiving, interpreting and implementing active ageing at the local organizational level (Ball 2019). This paper aims to help fill this gap, by describing one particular case study, against the backdrop of the framework agreement mentioned above.

The article explores two main issues. One is the narratives of social partners regarding the understanding of active ageing at the organizational level in Spain—a middle-ranked country in terms of its Active Ageing Index score (Walker 2019) and also the country with the second-highest unemployment rate in the EU at the time of research (Eurofound 2019). The second is the potential expedience and viability of implementing an active ageing policy—i.e. a policy that counters the tendency towards early retirement (Ebbinghaus and Manow 2001) and increases the ability of workers to stay in the labour market at least until the legal retirement age (CES et al. 2017)—in companies located in contexts of serious employment deficit. Following the approach of van Dyk et al. (2013), part of the purpose of this paper is to give a voice to a sample of Spanish line managers, workers and human resources managers and find out how closely they align with activation narratives put forward by the European Commission and European social partner representatives.

More specifically, the exploratory research presented below includes information from companies and other types of productive organizations (hereinafter companies) in four sectors in the most southern region of Spain with high unemployment on the following three issues: (a) how ageing and employment are perceived at the workplace; (b) what barriers and opportunities exist in relation to the adoption of an AA culture in local and regional companies; and (c) how employers and workers in the region are responding to top-down activation policies aimed at creating sustainable work opportunities for older workers—whether they come from the European Commission, European social partners or national authorities. Our expectation is that the “story lines” (van Dyk et al. 2013) of public narratives at international and national levels in favour of active ageing may be dissonant with social partners’ narratives in local organizations within challenging production environments. Such dissonance likely arises because the latter’s understanding of retirement and ageing well does not match institutional active ageing approaches that promote working life prolongation.

## Methods

The qualitative methodology of this study consisted of organizing “data-generating workshops” with workers and managers from four entrepreneurial organizations based in Andalusia, in southern Spain. In “data-generating workshops” (Patton 2015) participants attend with the intention of learning instead of feeling the pressure of being interviewed and the research questions serve as an artifact with which to guide the group conversation.

It was decided that the study should be performed in a region with high unemployment rates (21.83%) (EURES 2020) but in which policies promoting active ageing and employability of people of over 45 years old had been already implemented (Junta de Andalucía 2010).

### Procedure

First, a pilot data-generating workshop was held with the participation of 6 line managers and workers—who were also trade union representatives—from five companies in four different sectors. In the opinion of Creswell (2013), the use of 4–5 cases (i.e. companies) is most appropriate. The four sectors had been previously selected through purposive sampling aimed at guaranteeing sectoral diversity, due to the possibility that the characteristics of particular industrial sectors would have implications in the introduction of an active ageing culture—and might also affect the willingness of companies to participate. The company selection process also took into account criteria such as size, type of ownership (public/private), generational diversity and gender ratios.

A semi-structured workshop script was developed to address topics related to the three issues specified above, which were to be covered in the pilot workshop. This pilot workshop was audio-taped, transcribed and analyzed. In addition, to further the professional learning that, according to Patton (2015), can be obtained from this type of workshop, the participants were asked to answer, after the workshop and via email, four questions about active ageing in their companies. The results of the pilot workshop served to reconfigure the initial script and to prepare the issues to be addressed at subsequent workshops.

The four companies that agreed to continue participating in the research were invited to select from their staff a group of 3–5 individuals, including workers, managers and human resources managers. Then, four data-generating workshops—one per company—were held, each lasting two hours. Appendix A explains in detail some of the procedures used in the workshops. The condensed profiles of the participating companies are presented in Table 1.

**Table 1:** Condensed profile of participating organizations

	Workshop 1 (W1)	Workshop 2 (W2)	Workshop 3 (W3)	Workshop 4 (W4)
Sector	Industry	Education	Leisure, sports, tourism	Social health and social education services
Size	100–500 workers	Over 500 workers	100–500 workers	Over 500 workers
Ownership	Private	Public	Public	Private
Generational diversity	Average (no specific generational group predominates)	Low (heavy predominance of persons aged over 45)	Average (no specific generational group predominates)	Low (predominance of persons aged under 45)
Gender diversity	Mostly male employees	Male and female employees	Male and female employees	Mostly female employees
Participants (*n* = 20)	1 manager, 1 human resources manager, 3 workers’ representatives	1 manager, 2 workers’ representatives	1 manager, 1 human resources manager, 2 workers’ representatives	4 managers, 1 human resources manager, 3 workers’ representatives

A procedural and content protocol was developed and applied equally in all workshops. Furthermore, at the end of each workshop, the participants were invited to complete an assessment questionnaire about the experience. Throughout the process, the research followed the ethical standards established by the University of Newcastle (the study was part of a broader European project co-ordinated by this University).

### Analysis

The workshops were audio-recorded, transcribed verbatim and analyzed with NVivo 11. The analytic strategy implemented was mixed-methods, a combination of thematic inductive and deductive analysis through systematic coding carried out in six phases (Braun and Clark 2006). A draft codebook and an initial master list of codes were generated after the pilot workshop. Following several coding sessions, a final codebook with 85 parent codes and subcodes was reached. During the coding process, co-authors worked independently to pre-code, review and code the entire database in two cycles. Following Saldaña (2009), in the first cycle, structural, descriptive and In Vivo coding were applied, whereas pattern, focussed and axial coding to identify emerging categories were the main coding methods during the second coding cycle. Once both cycles had been completed, differences in coding were reconciled through discussion. Appendix B contains some examples of first cycle coding.

Regarding the assessment of inter-rater reliability, Cohen’s Kappa was used. In order to decide which value of Kappa would be acceptable, qualitative studies that had previously used this coefficient to explore active ageing were reviewed (Au et al. 2020; Kruse and Schmitt 2015). Finally, inter-rater reliability rate achieved was 0.82 (weighted Cohen’s Kappa by source size), a highly reliable value (Hruschka et al. 2004).

Once the preliminary report of the results had been drawn up, the researchers had additional meetings with workshop participants. The purpose of these meetings was to present the findings to the participants and correct potential misinterpretations made throughout the analytical process.

Overall, the study meets the criteria of trustworthiness and validity in accordance with established standards of qualitative inquiry in Gerontology (Cobb and Forbes 2002).

## Results

### Perceptions

As for the first explored issue—i.e. how questions related to ageing and employment are perceived in workplace contexts –, active ageing was understood to be something related to “ageing well”, but—with very few exceptions—participants were unable to go into detail. Lack of familiarity with the concept of active ageing coexisted with a very deeply ingrained idea; to age well you have to stop working as soon as possible:

You go through life, you start working and [ageing] is the last thing on your mind. You keep going and then a moment comes, I don’ really know at what age. You realize: this affects me. I think that before that moment, in other places as well, [ageing] is not relevant to you. It’s something that will never come. I think that here workers [do not think about ageing] until we reach that particular moment. The important thing, in the end, is the time you have left, the sooner you retire the better. (worker)

The question of age and ageing was not very present in the day-to-day activity of workplace contexts. The workshops offered an opportunity to reflect on the issue, as participants were invited to share their thoughts. Which aspects of the relationship between age and ageing were most often mentioned? Here is a sample:

- The value of experience, connected to the multiplicity of ages and the relationship between different generations at the company:
  Associating age with the issue of skills and competencies, understanding that it might be the people who are older in the company, in the service provided, that are better prepared to deal with certain issues that are more complicated or conflictive. (line manager)
- The diversity of jobs does not allow for a uniform response to the issue of ageing:
  In the end, what we’re moving towards is an attempt [to keep people working longer], since we are now living longer and longer. I believe that is the direction we are going, but I still think it depends on the work you do. (worker)
- Active ageing should be a voluntary option, to be chosen when the right conditions are in place:

How do we sell that idea [of extending people’s working life]? How can it be sold? It is very difficult, but if it must be done in the end, we will have to do it in a way that people feel motivated, they feel they are highly regarded. It has to be voluntary. […] this is a voluntary matter that cannot be imposed on anybody. (worker)

### Obstacles

The perceived obstacles to active ageing—included in the second explored issue, namely barriers and opportunities to adopt an AA culture in the company—were more organizational than individual in nature. They included, for example, questions related to the type of workforce management, lack of awareness about ageing in general—and active ageing in particular –, difficulties related to legal or social policy issues, trouble carrying out the necessary adaptation of jobs as workers grow older and, finally, certain aspects of human resource policies.

How could workforce management be an obstacle to the introduction of an active ageing culture? Primarily because of the difficulty of combining actions aimed at facilitating active ageing with those aimed at maintaining and improving production processes and service provision. This, in turn, was closely related to one of the themes most frequently mentioned by the participants: the adaptation of jobs as workers grow older.

Many of those agreements, on conciliation, adaptation…they are actually, hmm, incompatible, you might say. That is, if the measures are not applied very carefully, they are incompatible with providing a service with a certain degree of normality. (human resources manager)

Furthermore, a single company has multiple interests and they are often contradictory: the type of workforce management that is good for one sector of employees may not be ideal for other sectors. This problem could be more or less problematic depending on the number of people involved: how can a company properly manage an ageing workforce when it has a high number of employees, when the costs associated with any change in the form of management are high and when there is a wide range of employment contract types? All of these were common concerns.

Lastly, it was recognized that the management level as a whole faces considerable challenges related to the necessary training of employees and updating the workforce as employees grow older. For example, workshop participants admitted that it was not always easy to find the right person to partially replace an older worker who should work fewer hours to be able to continue to perform the job satisfactorily, in accordance with the ageing process. However, when no job adaptation process was implemented—that is, when no measures were introduced to adjust the job requirements to the real capabilities of the worker who is growing older –, the situation ended up moving in the opposite direction, away from active ageing; the worker was laid off or given permanent leave for health reasons. In some workshops, it was even pointed out that workers were afraid of being let go if they displayed some health problem or limitations in their capacity for good performance. Obviously, such a sentiment denotes the complete absence of a positive vision of ageing at the company:

That [the issue of ageing] is something we have never talked about at our company, only getting workers out of the way via early retirement, so we can get in some new people, which also works out much cheaper for the company and that, of course, is an added value. (worker)

In the organizational sphere, job adaptation was perceived sometimes as an obstacle and sometimes as a facilitator to the development of an active ageing culture. Why an obstacle? Firstly, in certain cases adaptation was perceived as a source of labeling and, in consequence, as the precursor of perhaps being laid off in a context of great labour instability, such as Andalusia. So, there were workers who appeared reluctant to begin the process of adaptation of their jobs. Secondly, the adaptation possibilities were limited by the demands of the productive process or service provision—for example, there were companies that could not get by without workers doing night shifts, and others that provided a service at the home of customers who were not always willing to collaborate in the introduction of the most appropriate adaptations. The problem was also that it did not seem feasible to adapt the jobs of everyone who needed it; therefore, the question arose of who would be left out and for what reasons?

A final reason for deeming job adaptation to be an obstacle was the fact that some workers believed they had no choice but to adjust to existing conditions—even if they were not the right ones for ageing well—because otherwise, they risked running into problems with the company. This alternative manner of adaptation led to passivity in daily activity, preventing people from explicitly tackling the demands associated with implementing an active ageing culture in companies:

What you do not want is to generate more problems, so you say “ok, I will adjust”. You do not generate more problems but that doesn’t mean they don’t exist, because they do. (line manager)

Another type of obstacle had to do with the legal context and social policy issues at the macro level—for example, national legislation regulating retirement and pensions. It should be noted that in general workshop participants had very limited knowledge of this context: some people had vague ideas, had read one or two documents or regulations but it was mostly perceived as something far away from ordinary business activity. As mentioned earlier, active ageing was not among their most serious concerns. In addition, businesses worried about who would pay for the possible costs of the active ageing measures—for example, when the reduction of hours worked by an older worker required another worker to be hired, or when older workers had more periods of medical leave –. Businesses admitted that, to their knowledge, political initiatives favouring partial, active or flexible retirement had not turned out to be satisfactory. Why not? Because there had been no pedagogical activity accompanying them, explaining what they were and their purpose and ultimate goal—i.e. lengthening working life –:

Partial retirements have not worked in our company. I mean, there was an option, a choice that had to be made. The employee had to either work the partial retirement time continuously or, for example, it was done in just two months every year. Those who chose to do it in two months a year, well, neither the work they provided to the company was acceptable nor was it satisfactory for them to have to come back. (line manager)

In terms of labour law and hiring regulations, the real financial cost of laying off employees—sometimes less than what it cost to continue employing a senior worker—could also be an obstacle to the prolongation of working life:

I have taken part in the collective bargaining negotiations at the provincial level […] and, really, I am telling you that the social partners do try to convey to those running the business that they should adopt some age-related adaptation measures, but they won't even consider it. Their argument is that laying people off does not have as high a cost as adaptation. (trade union representative)

Whether the company belonging to the public or private sector was another factor that aided in understanding why the company had more or less difficulty adopting active ageing policies. One participant commented that public sector companies are more rigid and limited in their options when it comes to measures related to, for example, recognizing a worker’ trajectory or considering different hiring possibilities, such as when and how:

A company in the public sector cannot set up measures that incentivize, for example, retirement, it cannot reward people for that. In short, it faces a series of limitations. (human resources manager)

Then there was the question of the public pension system in a society with high unemployment and a high level of job instability. This dilemma was mentioned frequently at the workshops: individuals who might benefit from being able to retire sooner—for health or other reasons—but could not do so because of the loss of purchasing power that would come with receiving a pension instead of their salary. This situation gave rise to a kind of forced active ageing which, far from fomenting a positive culture in this field, had placed the concept in the position of yet another method of oppression by political authorities:

A person is 67 and cannot retire because he or she has not been paying into the public pension system long enough to reach the minimum contribution required. The company finds itself in the situation of knowing that the person physically should not be working because it is hard work. But it understands that the person wants to continue working because with another year, another eight months, he or she will be entitled to the full retirement pension. So it tries to adapt the position to the extent possible. (human resources manager)

Faced with this situation, the discourse of some managers was the following: if legislators keep pushing back the retirement age, companies will find themselves more limited in their ability to find ways to reduce the working life of those who are in precarious health. According to workshop participants, it was as if governmental action in favour of active ageing had in itself become an obstacle to properly addressing the needs of those who, far from working more, needed to do just the opposite. In short, companies faced a wide range of situations that required a multiplicity of responses that current regulations did not offer.

The rest of the basic obstacles identified were related to human resources policies: rigidity in the types of employment contracts, difficulties in the social dialogue—lack of fluid communication with unions, in some cases –, absence of horizontal mobility or internal promotion systems that helped employees visualize an occupational career in the company, insufficient training and motivation among workers. It was not that human resources managers were trying to put obstacles in the way; rather, the obstacles were organizational factors embedded in daily management activities. But they were not just organizational in nature; they were also individual. For example, the low motivation of workers to consider extending their working life—in a context in which the predominant message had been, and to a certain extent still was, just the opposite –:

We have not fomented motivation. We have a collective agreement. The general retirement age is 65, but in our agreement, […] retirement is at 60, so starting at fifty-something you start to count, I have five years left, six years left. (line manager)

### Facilitators

Still within the second issue analysed—i.e. barriers and opportunities to adopt an AA culture in the company—but turning now to elements perceived as facilitators for the introduction of an active ageing culture, workshop participants described the following scenario: again, it was mostly organizational factors that played a role in fomenting an active ageing culture. Specifically, and in order of importance, the following issues were mentioned: progressive increase of awareness, adaptation of the job and working conditions—something deemed an obstacle when not done or when done in the wrong conditions, but when viewed from the other side it was clearly a facilitator—and workforce management—with measures such as the strengthening of the role of the older worker training younger workers, recognition of experience and promotion of older persons, educating the workers on the issue of active ageing, planning in regard to the age evolution of the workers, or the extension of employment contracts to improve the contributions paid into the public pension system –. In one case the importance of having a company culture that did not discriminate based on age was highlighted:

In this company, no one has ever, ever suffered age discrimination. Never. Quite the opposite, in fact. We have seen people aged over 45, 50 or 55 who had a problem, risk of social exclusion because they couldn’t find a job, and we gave them the chance. (human resources manager)

Without a doubt, the action most often perceived as favourable to active ageing was job adaptation. The four participating companies gave examples of what they had done in this regard:

There are areas in which there have been […] some type of meeting, some actions, especially positive actions, not simply concern about it, but actually coming up with formulas that make it possible to adapt this last stretch of the life of the worker, because obviously it must be adapted. (human resources manager)

Raising awareness about the issue was also considered a fundamental facilitator, a sort of lever with which to foment a culture of active ageing. In this regard, there seemed to be high receptivity not only to talking about the issue but also to looking for ways to prevent ageing from becoming a serious problem. Some companies seemed to know why it is so important to raise awareness about ageing in the work world:

Within 10 or 15 years the profile of workers is going to change completely and the company has to be prepared, we cannot adapt to it afterwards. (human resources manager)

### Response to activation policies

Regarding the third explored issue—i.e. how employers and workers in the region are responding to top-down activation policies—the obvious consequence of a low degree of awareness and familiarity with the issue was that the response by social partners to active ageing policies—issued at the international or national level—was practically non-existent. After the analysis, it was clear that workshop participants were just beginning to consider such policies.

What responses did our fieldwork find, as isolated and preliminary as they might be? Adaptation of the job to ensure, to the extent possible, better health conditions in the performance of the activity; preparation of reports about the occupational health of workers to see what type of job adaptation would be necessary—this response was more the result of concern about health maintenance than about ageing –; procedures to lower retirement age—partial retirement was a good example of this –; preferential attention to older people to combat the job exclusion they tend to suffer; improvement of employment contract conditions—going from part-time to full-time work, for example—so that workers of a certain age could increase their contribution to the public pension system and thus have a better pension when they retire; use of a special substitution contract when replacing a semi-retired worker; social action programmes promoting leisure activity and sports.

What measures should be taken, in the opinion of employers, workers and their representatives, to prepare for and better respond to the challenge of ageing in general and of active ageing in particular? These are the recommendations they had for policy makers: (a) find formulas by which to adapt the last stage of the working life of workers; (b) build consensus among the social partners regarding the need to pay attention to active ageing; (c) combine experience and youth for the benefit of all generations at the workplace; (d) raise awareness among companies that although laying off an older worker may be “efficient” and cost the company less, the losses are greater than the gains; (e) set up permanent working groups at the company to address the topic; (f) try to make the real age of retirement closer to the legal age, since the former tends to be lower than the latter; (g) study financing mechanisms with which to pay for the costs of promoting active ageing; and, above all, (h) improve communication and dialogue. As regards this last suggestion, one participant said:

[It is necessary to] actively listen to the needs of workers, according to their personal circumstances. (human resources manager)

Lastly, it is important to mention the strategy of recognizing the value and experience of older workers. Three of the four workshops touched upon this question. Participants acknowledged that it was good to give recognition to workers, as they grew older, for their contributions, their trajectory, their accumulated experience. Only a couple of companies had done something along these lines. Receiving recognition was linked to having the motivation necessary to continue forming part of the production environment, instead of retiring when the time came. Both factors—recognition and motivation—were in turn related to the good management of a multigenerational workforce. And it was precisely interaction among members of a multigenerational workforce that was deemed a facilitator, something that helped pave the way for training and recycling, which are necessary to ensure that workers can keep contributing throughout their working lives:

The expert worker or the older worker, once he realizes that he has to learn new stuff, that what he knows is now old, [it is good] if that person […] is recognized by the company. These people are our trainers in a way. They have that prestige and I think for these people it is very gratifying. (human resources manager)

## Discussion

In the development of strategies to promote active ageing in Europe, there is a ʻstructural lagʼ between demographic changes and the response by institutions, which take some time to introduce policies designed to bring necessary changes, to make active ageing a real and sustainable option (Walker and Zaidi 2019). This delay seems to be especially noticeable in the work sphere (Walker 2018), particularly in contexts of high unemployment and in relation to social partners. When moving from institutional formulation at the macro level—“story lines” in public discourse (van Dyk et al. 2013)—to organizational space—social partners’ narratives –, the “distance” between the proposed policies—extending working lives as much as possible (European Commission, 2012) and promoting and managing active ageing effectively (CES et al. 2017)—and actions that can actually be implemented becomes very evident. For example, such “distance” might be illustrated by the difficulty of combining active ageing measures with productivity goals in a context in which early retirement has been the norm. Again, it has been demonstrated that “supportive strategies at the meso level of stakeholders and particularly companies are crucial” (Naegele and Bauknecht 2019: 114) in efforts to make working life extension truly feasible. In fact, the results of the present study, which examines a group of Spanish social partners, show precisely this.

In all the companies involved in this case study, regardless of sector, type or size, the concept of active ageing remains poorly understood at both organizational and individual levels. Moreover, the idea of retiring as soon as possible continues to guide most individual plans for old age, as is the case elsewhere (Hofäcker 2016). It is safe to say that at the time of fieldwork the topic of active ageing was practically absent from the agendas of the companies involved in the research, despite efforts by the European Commission, the European social partnership and national authorities. In general, the analysis has identified an unfortunate combination of lack of information—although in the distance, beyond the national level, there are glimpses of a EU that is imposing active ageing but without the blessings of those who must implement it—and lack of support—there are few specific and viable instruments to use in a context where unemployment is prevalent. Overall, at the organizational level more obstacles to active ageing are perceived than facilitators. However, macro-institutional “story lines” tend to focus more on the latter, emphasizing the multiple advantages of active ageing as a “win–win solution” (van Dyk et al. 2013) and implementing measures to promote and manage active ageing (CES et al. 2017).

In this context, the study has confirmed that there is a low level of awareness regarding active ageing and the challenges and opportunities linked to demographic change, as pointed out in the framework agreement (CES et al. 2017). It is thus essential that awareness campaigns on active ageing be undertaken in production environments (Guaglianone and Ravelli 2016) but it is equally important to take labour market realities into account.

Is this lack of awareness among social partners specific to Spain? Certainly not. A comparative study examining data from Italy, Poland, Spain and the United Kingdom has concluded that the level of awareness regarding active ageing among social partners in these countries is modest or very low (Ball 2019). This should be considered clear evidence of the limited efficacy, for now, of active ageing policies and associated “story lines” developed using a top-down model (López-López and Sánchez 2020).

The predominant idea in the collective imaginary, in the Spanish case studied, is that the goal should be to do everything possible to work the shortest time possible, and workers confirm the late freedom narrative, i.e. the view of retirement as a well-deserved liberation from employment duties (van Dyk 2015). Therefore, the most immediate response by employers to the ageing of employees has been to create formulas by which to encourage early retirement, even though the legislation—which, by the way, most people know little about—goes precisely in the opposite direction (Alcover et al. 2014).

Clearly, when it comes to trying to introduce an active ageing culture in companies located in a challenging production environment, obstacles abound. Promoting active ageing is, in general, not perceived as an easy thing to do: it seems it is difficult to find the type of workforce management that is right for the company and that also allows the needs of older employees to be met. In fact, the only substantial practice carried out to date in relation to active ageing has been the attempt—more or less elaborate and successful, depending on the case—to adapt certain jobs, although this initiative has sometimes been taken as a response not to ageing but rather to poor health or the inability of the workers to continue doing their assigned tasks. Interestingly, although it is often unconscious, this improvement of individual working conditions may help reduce early retirement (Moreira et al. 2018).

As regards the facilitators of active ageing, a two-tiered connection—between older workers’ recognition and their motivation to extend their working lives, on the one hand, and between intergenerational collaboration and the interest shown by older workers in training younger workers, on the other—has been identified. The second part of the connection suggests that it might be beneficial to add an intergenerational aspect to collective bargaining and social dialogue (ter Haar and Rönnmar 2014) and to set up viable systems for lifelong learning at the workplace (Carlstedt et al. 2018).

Finally, providing information and raising awareness is not enough. It is also necessary to implement realistic and pragmatic strategies that are suited to the situation of each company. Such strategies must effectively respond to the real needs of an ageing workforce and reduce existing obstacles and resistance, and they must do so without distorting the system of production or service provision in any way (Peña-Casas 2017).

Although the Spanish companies involved in the study have on certain occasions made decisions related to the ageing of their workers, some of the premises upon which these decisions were based are totally contrary to institutional “story lines”. It appears that, in the context of southern Spain, at this point in time, convincing employers and workers of the value of the prolongation of working life poses quite a challenge, particularly because what has been done up to the present is quite the opposite.

However, thanks to their participation in this research, some of the organizations involved are beginning to look at this issue and have added it to their agendas. Future research should take into account the effectiveness of fieldwork formats like data-generating workshops in raising dialogue about the impact of ageing within companies and also in eliciting bottom-up proposals and responses to public policies and measures in the field of active ageing.

### Limitations

The main limitation of this study is the small number of entrepreneurial organizations that agreed to be involved, although they do belong to four key sectors in the regional economy. In a context of very limited awareness at the organizational level about ageing in general and active ageing in particular, companies were reluctant to invest time in a research project that did not seem very relevant. However, the heterogeneous profile of the companies that did agree to participate at least allowed for a diverse range of narratives, providing tips and potential avenues for future research in this or other challenging production environments. Also, in future research, it would be a good idea to use a more diverse sample of social agents in terms of ethnicity and work history.

The fact that all the fieldwork took place in Spanish and was later partially translated into English for publication could be interpreted as a possible limitation. To ensure that no significant content was lost and that the English used was both correct and appropriate, the services of a professional translator with experience in the field of active ageing were engaged.

As for the transfer of the analysis to other European contexts, both the procedure and the results can be considered applicable elsewhere, given that all the research was conducted and validated step by step, in a parallel manner, in United Kingdom, Italy, Poland and Spain, although this paper focuses only on the last country. The final report of the larger project (Ball 2019) compares and contrasts the studies conducted in the different countries but, due to space limitations, such information could not be included here.

## Conclusion

Promoting active ageing in companies or other production settings does not consist only of prolonging working life, especially when an early retirement culture has prevailed in a context of high unemployment. It also involves developing realistic mechanisms that, taking into account the many obstacles, may facilitate job adaptation and adjustment by the worker during his or her final stage at the company. To this end, social partners at the local organizational level have a critical role to play, especially in challenging production environments such as the ones analyzed here. Despite their positive “story lines”, top-down active ageing policies—including European social partner agreements—alone are far from effective in counteracting the trend towards early retirement that may exist at the local level.

Information and measures exist but they must be interpreted and transposed to the real circumstances of companies and of society as a whole, through adequate ad hoc policies. This is the only way that employers, workers and their representatives—particularly in contexts of high unemployment—will come to be aware of and sensitive to the active ageing of workers and the need to take additional steps to improve the quality of life of workers as they age.

## Supplementary Information

Below is the link to the electronic supplementary material.

Supplementary file1 (DOCX 17 KB)

Supplementary file2 (DOCX 19 KB)

## Data Availability

Data and materials are available from authors upon request.
