# Supplementary material for: Active ageing policy in challenging production environments: a case study involving social partners in Spain
Source: Eur J Ageing. 2021 Sep 24;19(3):509–19. doi: 10.1007/s10433-021-00650-6 (PMC9424458; doi:10.1007/s10433-021-00650-6)
Supplement: Supplementary file 1 — Supplementary file1 (DOCX 17 KB) [file 10433_2021_650_MOESM1_ESM.docx]

**Supplementary Material**

Appendix A: Workshop implementation

All data-generating workshops began with a brief introduction of each person participating (name and position in the organization) and an explanation intended to clarify the aims of the research project and its three central concepts: active ageing, industrial relations and social dialogue. Then the purpose of the workshop itself was explained: to learn and share information and opinions regarding active ageing in the organization. All in a workshop lasting two hours. It was also explained that the results of the workshop would be used to develop training materials about active ageing that people from the participating organizations could use free of charge. After this beginning phase, each workshop was structured into six parts, each one consisting of a predetermined activity.

In the first place, participants were informed – verbally and in writing –, about the informed consent form and they were invited to sign it as a necessary step prior to participation in the workshop.

The longest part of each workshop consisted in discussing, one by one, the 6 key questions identified in the pilot workshop. To facilitate discussion and to preserve the learning dimension characteristic of this type of workshop, the dialogue began with a review of the terminology about active ageing and labour relations that had been used in the pilot workshop. This allowed the participants in each workshop to better grasp the questions that would be discussed subsequently. Next, each of the six following questions was put forward for discussion: 1) how is the issue of age and ageing perceived in the day-to-day activity of the organization; 2) how could the labour relations at the organization facilitate or hamper collective agreements about active ageing; 3) how are the unions and the organization responding to European Union and national active ageing policies; 4) how does the organization reconcile the interests of older workers to those of younger workers; 5) what best practices in active ageing have been used in the organization, if any, and 6) what type of materials would be most suitable for training in the area of active ageing at the organization.

In the third place, and as a complement to the preceding group activity, participants were invited to do an individual written activity: to identify the 3 key issues affecting active ageing in the organization, the 3 most important obstacles to the introduction of an active ageing culture and the 3 elements that could best facilitate said active ageing culture.

In the fourth place, the workshop participants were invited to take part in a future session for the validation of the training materials to be elaborated following the analysis of all the information obtained in the four workshops.

In the fifth part of the workshop, the participants were asked to answer a short evaluation of the experience, consisting of 5 Likert-type questions (e.g., Do you think it likely that in the future you will talk about active ageing with the people you work with?) and two open-ended questions (e.g., What part of the workshop has seemed the most useful to you?).

And finally, the participants were invited to record, right then, a mini-video lasting one minute in which they responded to one of the five first questions discussed in the workshop. These videos would later be used as an audiovisual resource in the training materials.
