# Supplementary material for: Active ageing policy in challenging production environments: a case study involving social partners in Spain
Source: Eur J Ageing. 2021 Sep 24;19(3):509–19. doi: 10.1007/s10433-021-00650-6 (PMC9424458; doi:10.1007/s10433-021-00650-6)
Supplement: Supplementary file 2 — Supplementary file2 (DOCX 19 KB) [file 10433_2021_650_MOESM2_ESM.docx]

**Supplementary Material**

Appendix B: Examples of first cycle coding

**Structural coding**

Sample research question: How do different industrial relations structures facilitate and/or inhibit the dissemination and implementation of collective agreements on active ageing?

Structural Code: Obstacles related to age

- Participant (P)#1: Well, yes, there are obstacles because there are people who at a certain age would like to retire early but the working conditions that you have when you take an early retirement are not fair.
- P#2: For example, a person is 67 and cannot retire because he or she has not been paying into the public pension system long enough to reach the minimum contribution required. So the company finds itself in the following situation; it knows that this person should not be working because the work is hard, but it understands perfectly well that the person wants to keep working because within a year, or eight months, he or she will have a good retirement pension, so the company tries to adapt the job to the extent possible. This does not change the fact that the person, because it’s true that his or her capacities aren't what they used to be, has a greater risk of a workplace accident.

**Descriptive coding**

| P#1: Well, yes, there are obstacles because there are people who at a certain age would like to retire early but the working conditions that you have when you take an early retirement are not fair.  P#2: For example, a person is 67 and cannot retire because he or she has not been paying into the public pension system long enough to reach the minimum contribution required. So the company finds itself in the following situation; it knows that this person should not be working because the work is hard, but it understands perfectly well that the person wants to keep working because within a year, or eight months, he or she will have a good retirement pension, so the company tries to adapt the job to the extent possible. This does not change the fact that the person, because it’s true that his or her capacities aren't what they used to be, has a greater risk of a workplace accident. | OBSTACLE  EARLY RETIREMENT  WORKING CONDITIONS  WORKING CONDITIONS  ADAPTATION  RISK |
| --- | --- |

**In Vivo coding**

| P#3: They are not internal obstacles.  P#4: Are we talking about external obstacles?  Workshop facilitator: You can talk about internal and external obstacles.  P#5: In my view, external obstacles exist. As for internal ones, I think that nowadays there are none that I would identify as such. It could be that in the future the organization will generate them. | “INTERNAL OBSTACLES”  “EXTERNAL OBSTACLES”  “EXTERNAL OBSTACLES”  “INTERNAL OBSTACLES” |
| --- | --- |
